# Components of Family-Focused Interventions that Have Common Impacts Across Parental Domestic Violence and Abuse, Mental Ill-Health, and Substance Misuse: An Intervention Components Analysis

**DOI:** 10.1007/s10935-025-00868-5

**Published:** 2025-09-01

**Authors:** Kate Allen, Tamanna Malhotra, Amy Bond, Alice Garrood, G. J. Melendez-Torres, Tamsin Ford, Chris Bonell, Vashti Berry

**Affiliations:** 1 University of Exeter, South Cloisters, St Luke’s Campus, College Road, Exeter, EX1 1TE UK; 2 University of Birmingham, Edgbaston, Birmingham, B15 2TT UK; 3 Department of Psychiatry, Herchel Smith Building, Forvie Site, Robinson Way, Cambridge, CB2 0SZ UK; 4 London School of Hygiene and Tropical Medicine, 15-17 Tavistock Place, London, WC1H 9SH UK

**Keywords:** Intervention components analysis, Domestic violence and abuse, Mental ill-health, Substance misuse, Family, Prevention

## Abstract

Support for families experiencing domestic violence and abuse (DVA), mental ill-health (MH) and substance misuse (SU) is often delivered in siloes, despite the frequent co-occurrence of these public health issues. Little evidence-based guidance exists on which interventions best support families experiencing a combination of these problems. Identifying intervention components with common impacts across parental DVA, MH and SU could inform policy and practice. We conducted an Intervention Components Analysis (ICA) to identify intervention components that have common impacts across parental DVA, MH and SU. We searched ten databases for randomised controlled trials of family-focused interventions targeting, and measuring an impact on, one or more of these issues. We developed an initial coding framework using open coding to guide the coding of subsequent studies. Descriptive analyses identified common components across target outcomes (DVA/MH/SU) and robust variance meta-regressions explored the relationship between intervention components and treatment effects. A Lived Experience Advisory Group informed our presentation and interpretation of the results. We identified 164 interventions: 40 focused on a combination of DVA, MH and SU and 124 addressed one issue alone. None of the 20 components identified were unique to any specific outcome and no single component was associated with meaningful improvement in outcomes. Interventions aiming to provide integrated support across outcomes were less successful at improving MH and SU outcomes than those targeting single issues. We found no evidence of commonly effective intervention components. Better alignment between components and underlying processes driving DVA/MH/SU, and alternative intervention designs, are needed.

## Introduction

Children who experience parental domestic violence and abuse (DVA), mental ill-health (MH) and substance misuse (SU) are at greater risk of various adverse outcomes during childhood and later in life (Cleaver et al., 2011; Hughes et al., 2017; Kelley et al., 2015; Rodriguez, 2006). These adverse childhood experiences (ACEs) are prevalent public health problems in the UK (Baker, 2021; Office for National Statistics, 2022a, 2022b) and worldwide (United Nations Office on Drugs and Crime, 2021; WHO, 2018, 2021, 2022). In the UK, approximately 5% of the population have experienced DVA victimisation (Office for National Statistics, 2022b), 17% have experienced MH (Office for Health Improvement and Disparities, 2022), 11% have experienced increasing risk or higher risk alcohol drinking behaviour (NHS Digital, 2024), and 9.2% have used illict drugs in the past year (Office for National Statistics, 2022b). Notably, an estimated 3.6% of UK families are likely to experience these ACEs in combination (Chowdry, 2018). Families experiencing this co-occurrence or clustering of ACEs are likely to be particularly vulnerable because services are ill-equipped to address the complexity of multiple needs (Allen et al., 2024; Kedzior et al., 2024; Mason & O’Rinn, 2014). Increasing our understanding of how best to support these families is essential (HM Government, 2017, 2019; Safe Lives, 2019).

There is some evidence about how to address parental DVA, MH and SU when considered individually. Previous systematic reviews have identified that interventions focused on intensive advocacy (British Columbia Centre of Excellence for Women’s Health, 2013; Rivas et al., 2015) or empowerment (Kiani et al., 2021) can be beneficial for women experiencing DVA, and there is some evidence that interventions involving motivational interviewing or behavioural couples therapy might reduce perpetration of abuse (British Columbia Centre of Excellence for Women’s Health, 2013). Those that include components targeting emotional regulation skills, through addressing thought-emotion-behaviour cycles (e.g., cognitive behavioural therapy) or improving interpersonal relationships (e.g., interpersonal therapy), are well known to be effective in treating MH problems such as depression (Cuijpers et al., 2013; Dennis & Dowswell, 2013) and anxiety (Bandelow et al., 2017). Support that includes intensive case management or involves family members or a parenting component can help reduce parental SU (McGovern et al., 2018, 2021), as well as psychoeducation, social support, developing action plans and goal setting (Gomez et al., 2021).

In contrast, our understanding of what works to support families with multiple, co-occurring needs related to DVA, MH and SU are limited because services and research mostly exclude those with more than one of these difficulties. Recent systematic reviews found few evidence-based interventions addressing such needs. In a large review of reviews, Barrett et al. (2024) identified only two reviews that synthesised evidence on interventions aiming to provide support for children exposed to multiple ACEs including parental DVA, MH and SU (Courtin et al., 2019; Marie-Mitchell & Kostolansky, 2019), and no reviews of interventions aiming to address all three. Our recent systematic review addressed this gap, reviewing family-focused interventions addressing parental DVA, MH and SU. However, we found only one study reporting benefits for two of these outcomes and no interventions reporting benefits for all three (Allen et al., 2022).

In the absence of effective evidence-based interventions, we need to consider what an intervention for parental DVA, MH and SU should look like. Alongside engaging with families, service providers and senior leadership (Allen et al., 2024; Muir et al., 2024), one way to do this is to identify common intervention components across interventions addressing parental DVA, MH and SU, and consider which components might have common impacts across parental DVA, MH and SU (Allen et al., 2022; Barrett et al., 2024). Highlighting key components that are hypothesised to contribute to changes in parental outcomes is essential to identify which aspects of support are most helpful for co-occurring parental DVA, MH and SU. Furthermore, understanding which common components have demonstrated success in addressing parental DVA, MH and SU will help guide the development of future interventions, and provision of current services. To our knowledge, no previous studies have explored this despite an identified need within the literature (Allen et al., 2022; Barrett et al., 2024).

This study aims to identify the ‘best-bet packages’ for parents at risk of, or experiencing, a combination of DVA, MH and SU, with a focus distinct elements of intervention content or design described and hypothesised to contribute to change in key outcomes. This deeper analytical approach will help inform the development of new interventions targeting these clustered outcomes and guide provision of services. It will also highlight any adverse effects of specific components or those that may warrant additional investigation.

## Materials and methods

This intervention components analysis (ICA) extends a systematic review we conducted to examine the effectiveness of family-focused interventions for parental DVA, MH and/or SU (Allen et al., 2022). While some of the methods have been previously reported (Allen et al., 2022), we briefly summarize these below and expand to outline the methods unique to the ICA. The protocol for both the ICA and systematic review are available on PROSPERO (CRD42020210350).

### Eligibility Criteria

We included studies that: employed a randomised controlled trial (RCT), cluster RCT or pilot RCT; focused on parents/carers at risk of, or experiencing, one or more of parental DVA, MH and SU or children in their care; involved a psychosocial family-focused intervention; and measured one or more of DVA (restricted to victimisation/perpetration (HM Government, 2019), MH (restricted to common mental health disorders including depression, anxiety, post-traumatic stress disorder (PTSD), panic disorder, obsessive compulsive disorder (OCD) and general mental health of parents/caregivers (NICE, 2011) or SU (restricted to alcohol or drug use) of parents or caregivers. We focused on common mental health disorders as these commonly co-occur with DVA and MH and allowed us to ensure the search results were manageable. We excluded studies focused on postnatal depression as this is restricted to a specific time period and disorders such as bipolar depression, psychosis, schizophrenia and borderline personality disorder as these are considered severe mental-health disorders.

### Search Strategy

Our search was developed with support from an information specialist (Alison Bethel, University of Exeter) and was conducted by KA. We searched the following databases from inception to March 2020 and updated searches in July 2021: MEDLINE, PsycINFO, Embase, CINAHL, Education Research Information Centre (ERIC), Sociological Abstracts, Applied Social Sciences Index & Abstracts (ASSIA), ProQuest Dissertations and Theses Global, Web of Science Core Collection and Cochrane Central Register of Controlled Trials (CENTRAL). Our search terms fell into five main categories, combined as follows: [1) DVA OR 2) MH OR 3) SU] AND 4) parents/family AND 5) RCTs. Our search included free-text and controlled search terms, adapted for each database. All searches were limited to ‘English Language Only’.

### Study Selection

We imported search results to EndNote V9 (The EndNote Team, 2013) and removed duplicates. We used EPPI-Reviewer 4 RCT classifier (EPPI-Reviewer, 2015) to categorise the results based on likelihood of pertaining to an RCT. Studies classified as < 20% likelihood of pertaining to an RCT were title-and-abstract screened by KA alone. Studies classified as ≥ 20% likelihood of pertaining to an RCT were title-and-abstract screened by KA and a second independent reviewer. All full-text screening was conducted by KA and 10% were screened by a second independent reviewer. Agreement levels were > 90% and therefore no further second screening was conducted. Any disagreements were resolved through consultation with a third reviewer (VB).

### Data Extraction

TM, AB and KA extracted information from included studies using a standardised form piloted prior to use (Supplementary File 1) including study design, participants, intervention details, outcome measures and intervention/control group raw scores (including N, mean, SD, SE, CI, and p-value) at baseline, post-intervention and the latest possible follow-up time point. Outcome measures included self-report or validated measures of: DVA (including physical, sexual, psychological/emotional, controlling/coercive behaviour and economic abuse (HM Government 2019)) victimisation, perpetration and victimisation/perpetration; depression, anxiety, panic disorder, PTSD, OCD and general MH; and alcohol use, drug use (including specific types of drug use) and general SU. We did not extract information on proxy measures of these outcomes.

We extracted all descriptive text outlining the intervention from the included papers and any relevant, referenced supplementary material. This included text from introductions, methods and discussions. We did not conduct forward or backward citation searches for additional information on the interventions or contact study authors.

### Quality Appraisal

TM, AB and KA quality-appraised studies using the Risk of Bias Tool 2 (RoB2) (Higgins et al., 2019; Eldridge et al., 2020). The RoB2 examines potential sources of bias related to the randomisation process, participant identification and recruitment, intervention assignment, handling of missing outcome data, outcome measurement and the reporting of results (Higgins et al., 2019; Eldridge et al., 2020).

### Data Analysis

We used Sutcliffe et al.’s (2015) approach to undertake our ICA. This involved developing an understanding of the differences between interventions, and using this information to assess differences in outcomes both within and across conditions (Sutcliffe et al., 2015).

#### Developing an Understanding of the Differences between Interventions

TM and KA open-coded the same random selection of 30 studies to generate an initial intervention-components framework. In our ICA, we define ‘intervention component’ as a distinct element of intervention content or design that was described and hypothesised to contribute to change in key outcomes—namely, parental experiences of DVA, MH and/or SU. These may pertain to intervention content, delivery processes, relational or contextual adaptations, or support mechanisms. As such, we focused on coding intervention components that told us something about *how* the intervention might work (e.g., intervention strategies, frameworks or overarching theories) rather than intervention descriptors (e.g., length of the intervention, number of sessions etc.), which were extracted elsewhere. This initial framework was then used by TM, AB, AG and KA to continue coding other included studies while allowing for flexibility to generate new codes as needed. The team met regularly to discuss coding, agree additional codes and adapt existing codes based on the literature. Once all coding was completed and the framework finalised, coding of the included studies was checked by AB and KA to ensure that any new codes had been applied consistently across studies. All coding was conducted in NVivo and, at the end of the process, an Excel table was produced to highlight the intervention components present across each study. We descriptively analysed the components commonly present across DVA, MH, SU and clustered (targeting two or more of DVA, MH and/or SU) interventions.

#### Assessing Differences in Outcomes

We conducted random-effects meta-regressions (Borenstein et al., 2007) with robust variance estimation (Tanner-Smith & Tipton, 2014; Tipton, 2015) to examine the effect of family-focused interventions and intervention components on parental DVA, MH and SU at post-intervention and later follow-up. First, we used meta-regressions to examine differences in outcomes at post-intervention and later follow-up based on the identified intervention components, examining patterns across outcomes to highlight which components have common beneficial or harmful effects across outcomes. Second, we examined the effect of single-issue interventions (targeting one of DVA, MH or SU) and clustered interventions (targeting two or more of DVA, MH and SU) on parental DVA, MH and SU outcomes at post-intervention and later follow-up. Here, we grouped dyadic (targeting two of DVA, MH and SU) and clustered interventions (targeting all three of DVA, MH and SU) together for simplicity. We did not examine how contextual factors – such as fidelity, participant engagement or setting – may shape the effectiveness of the identified components, nor potential synergistic or interactive effects between components. However, all trials were subject to an RCT (typically with high fidelity). While contextual considerations are important, they were beyond the scope of the current analysis.

#### Patient and Public Engagement and Involvement

Patient and public engagement and involvement (PPIE) with commissioners and service providers shaped the design and focus of our ICA, informing our focus on family-focused interventions and our decision to include prevention and treatment interventions to ensure our findings would be relevant for providers and commissioners (Allen et al., 2022). We also held two PPIE workshops with an established Lived Experience Advisory Group to help inform our interpretation and presentation of the ICA findings. This group included five people with experience of violence and abuse, MH and SU (https://www.vamhn.co.uk/lived-experience-advisory-group.html). The first workshop focused on familiarising participants with the ICA and reviewing the preliminary intervention components table. The second workshop involved discussing the key ICA findings in greater depth. Both workshops lasted two hours and were structured around guided prompts to elicit the groups reflections on: the naming and descriptions of components, common/uncommon components, key findings and implications. We also consulted five service providers about the results of the study in a one-off workshop which involved reflecting on the findings and key points raised by the Lived Experience Advisory Group workshops. We have acknowledged where these groups have contributed to our presentation/understanding of the findings throughout.

## Results

**Fig. 1:**
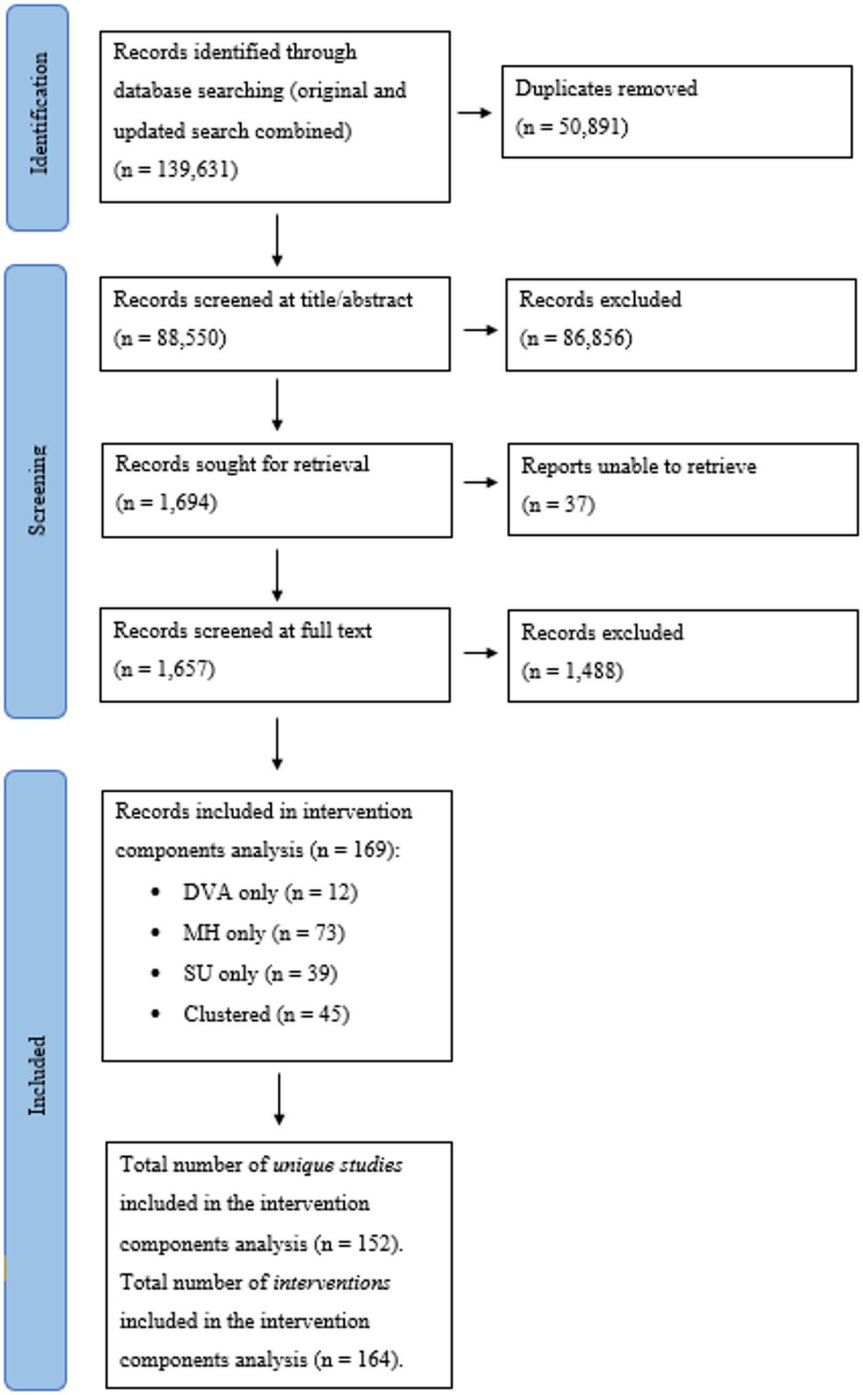
Number of records identified, screened, and included in the ICA

Figure 1 reports the number of records identified, screened and included in the ICA. Our searches retrieved 88,550 unique references. Of these, 1657 were screened at full text. Full-text screening resulted in 169 records included in the ICA: 12 records represented studies that measured parental DVA, 73 measured parental MH, 39 measured parental SU 27 measured two of parental DVA, MH and/or SU and 18 measured all three of parental DVA, MH and/or SU.

Of the 169 records included in the ICA, 152 unique studies were identified (some records referred to the same study). These studies reported on 164 interventions which were included in our analyses (some studies reported on two or more interventions). Using the RoB2, the majority of studies were rated as either ‘some concerns’ or ‘high risk’ of bias (see Supplementary File 2). From this point onwards, we refer solely to the 164 interventions included in our analysis. We refer to studies measuring DVA only as ‘DVA interventions’, MH only as ‘MH interventions’, SU only as ‘SU interventions’, two of DVA, MH and SU as ‘dyadic interventions’, and all three of DVA, MH and SU as ‘clustered interventions’.

### Characteristics of Included Interventions

All interventions aimed to improve the outcomes of parents and families broadly, but interventions primarily targeted either mothers, fathers, the caregiver, the child or the whole family. Of the 164 interventions reported, 71% focused on the mother, 16% on the family, 7% on the caregiver, 4% on the father and 2% on the child. In terms of level of prevention, 43% of the interventions intended to treat participants, 35% had multiple levels of prevention, 12% were secondary prevention and 9% were tertiary prevention.

Of all the interventions, 9% targeted DVA, 43% targeted MH, 24% targeted SU, 16% were dyadic and 8% were clustered. Although the review included interventions tested across 23 countries, interventions were most commonly undertaken in the U.S. (65%), the U.K (6%) and Australia (5%). Of the U.S. interventions, 34% targeted MH, 28% targeted SU and another 29% targeted dyadic or clustered risks. Only 9% of U.S. interventions primarily targeted DVA. A similar distribution was observed for U.K and Australian interventions, with most targeting MH, followed by SU and DVA. Interventions were also undertaken in Bosnia and Herzegovina (0.6%), Canada (4.3%), China (0.6%), Columbia (1.2%), Germany (0.6%), Greece (1.2%), Guatemala (0.6%), Iran (1.8%), Jamacia (0.6%), Jordan (0.6%), Nepal (0.6%), Netherlands (2.4%), New Zealand (0.6%), Pakistan (2.4%), Peru (0.6%), Romania (0.6%), Rwanda (1.2%), South Africa (1.8%), Sweden (0.6%) and Thailand (0.6%). Full details of intervention characteristics are provided in Supplementary File 3.

### Intervention Components Analysis

Our initial intervention-components framework involved 99 codes (Supplementary File 4). Of these, 12 codes fell under the broad category of intervention ‘activities’ and 87 codes under ‘pathways to change’ i.e., *how* interventions were attempting to reduce or prevent parental DVA, MH and/or SU. Continued coding of intervention descriptions and regular team discussions refined the initial framework into 26 codes. This revised framework was applied to the remaining intervention descriptions and further refined following extensive discussion to produce a final framework of 20 codes (Table 1). Four of these contained additional sub-codes describing additional intervention details. Supplementary File 5 describes the most common components identified.

**Table 1:** Intervention components identified including prevalence of component overall and for DVA, MH, SU, dyadic and clustered interventions, and PPIE rated priority level

Intervention component		PPIE Priority level	% (*N*)						Brief description of component and example of coding
			Overall	DVA	MH	SU	Dyadic	Clustered	
1. Addressing additional needs		**1**	18% (30)	14% (2)	10% (7)	26% (10)	19% (5)	46% (6)	Components which addressed additional needs during the intervention such as economic, housing and employment needs: *“Particular emphasis is placed on providing services for mental health*,* substance abuse*,* trauma*,* and other pertinent support and treatment needs along with practical services such as links to child care and employment linkages*,* and assistance with applying for benefits. FCTI case managers work closely with social service case managers to facilitate connections to resources through homeless services and community agencies.”* (p. 522, Samuels et al., 2015)
2. Changing cognitive processes		**2**	32% (53)	7% (1)	37% (26)	28% (11)	48% (13)	15% (2)	Components which focused on changing maladaptive thought process (i.e., CBT): *“Cognitive therapy components for the treatment of depression were integrated into each session. […] Mothers were encouraged to increase the number of personally reinforcing family activities*,* and the principles of the cognition-behavior-emotion cycle were explained to enable mothers to understand the linkages among mood*,* thinking*,* and behavior toward their children.”* (p. 99, Sanders & McFarland, 2000)
3. Community referrals		**3**	18% (30)	36% (5)	15% (11)	18% (7)	11% (3)	31% (11)	Components which linked participants with organisation and charities in the community to aide and support their DVA, MH or SU: *“Help families access to community resources*,* including services to address risks such as domestic violence*,* parental substance abuse*,* and poor mental health”* (p. 626, Duggan et al., 2004)
4. Cultural and community adaptations		**1**	17% (28)	7% (1)	27% (19)	10% (4)	11% (3)	8% (1)	Interventions that modified elements to recognise and reflect different perspectives, needs and practices of different at-risk groups, cultures and communities: *“Using descriptive data from symptomatic low-income mothers*,* the intervention was tailored to fit low-income mothers by organizing the four IPT foci around problems they typically faced (Beeber*,* Perreira*,* & Schwartz*,* 2008). The content for each interpersonal area was distilled into several one-page modules written in the vernacular that mothers used to describe their interpersonal problems. In each IPT module*,* specific strategies were introduced as the means to resolve the problems (Beeber et al.*,* 2004). The modules were written using words that mothers with limited literacy could read.”* (p. 5, Beeber et al., 2013)
5. Developing a safe intervention space			42% (69)	71% (10)	37% (26)	31% (12)	52% (14)	54% (7)	Components which emphasise the importance of developing a safe and comfortable intervention space.
5 A. Intervention environment		**1**	17% (28)	50% (7)	15% (11)	13% (5)	15% (4)	8% (1)	Components which specifically details considerations and changes to the intervention: *“Although traditionally provided in clinic settings*,* flexibility of delivery site (home vs. clinic) was offered to reduce the possible stigma associated with receiving mental health services for low-income*,* racially and ethnically diverse participants and to increase receptivity to services.”* (p. 605, Handley et al., 2017)
5B. Relationship between participant and interventionist		**1**	34% (56)	43% (6)	31% (22)	18% (7)	52% (14)	54% (7)	Components which support the development of strong and trusting participant-interventionist relationships: *“In terms of therapeutic characteristics*,* four features define RPMG as an intervention. The first is a supportive therapists’ stance. Encompassing the Rogerian constructs of acceptance*,* empathy*,* and genuineness*,* this is essential to foster a strong therapeutic alliance and to meet mothers’ unmet developmental needs”* (p. 244, Luthar et al., 2007)
6. Developing healthy relationships			59% (97)	50% (7)	72% (51)	31% (12)	63% (17)	77% (10)	Elements of the interventions which targeted the enhancement of relationships between family members.
6 A. Family		**2**	23% (37)	14% (2)	27% (19)	10% (4)	19% (5)	54% (7)	Improved elements of relationships within the family unit, between and across relatives: *“The IFSP is a written plan between the family and the home visitor that assists them in setting achievable goals to alleviate family stress and to enhance aspects of parental and family functioning.”* (p. 804, Duggan et al., 2007)
6B. Parent-child		**1**	44% (72)	21% (3)	61% (43)	15% (6)	48% (13)	54% (7)	Improved relationships between parent and child: *“The skills were presented in sequence; the initial skills focused on improving the quality of the mother–child relationship and increasing prosocial child behavior*,* and the latter skills focused on reducing problematic behavior”* (p. 709, Jouriles et al., 2009)
6 C. Parent		**1**	13% (21)	36% (5)	10% (7)	8% (3)	11% (3)	28% (3)	Improved interparental relationships: *“The class series was implemented as four prenatal classes*,* which serve to introduce the couple to themes and skills around strengthening their relationship*,* co-parenting*,* and parenting at the transition to parenthood”* (p. 971, Kan & Feinberg, 2014)
7. Empowerment		**2**	14% (23)	36% (5)	10% (7)	13% (5)	19% (5)	8% (1)	Element of an intervention which focused on supporting an individual to recognise their strengths and capabilities and which aimed to support participants to take control of their actions and lives: *“The intervention was based on an empowerment protocol developed by Parker et al. (13) designed to enhance abused women’s independence and control. It consisted of advice in the areas of safety*,* choice making and problem solving.”* (p. 1251, Tiwari et al., 2005)
8. Enhancing motivation to change		**2**	26% (43)	14% (2)	13% (9)	51% (20)	26% (7)	38% (5)	Techniques used to encourage and promote one’s motivation to change their behaviour: *“This study examined the usefulness of an MII [Motivational Interviewing Intervention] to decrease alcohol use during pregnancy […] MI [Motivational Interviewing) is a directive*,* patient-centered counseling style that assists persons in the exploration and resolution of ambivalence related to behavior change*,* which enhances motivation for change”* (p. 436, Osterman & Dyehouse, 2012)
9. Enhancing Skills			88% (145)	79% (11)	97% (69)	77% (30)	85% (23)	92% (12)	Any aspects of the intervention which targeted the development or enhancement of specific skills.
9 A. Communication skills		**1**	21% (35)	36% (5)	18% (13)	23% (9)	22% (6)	15% (2)	Enhanced the development of the participants ability to talk, share and express their ideas and emotions: *“The relationship education portion teaches couples positive communication skills and also teaches about negative communication styles such as invalidation and withdrawal.”* (p. 260, Wadsworth et al., 2011)
9B. Conflict resolution		**1**	10% (16)	50% (7)	10% (7)	3% (1)	4% (1)	0% (0)	Supported participants to understand and undergo the process of find peaceful solutions to disputes: *“The nurses also helped both partners to make safer decisions for the sake of themselves and their child*,* such as preventing arguments from escalating to a physical fight by teaching them how to address these situations*,* and by teaching them how to negotiate and to listen to each other”* (p. 3, Mejdoubi et al., 2013)
9 C. Coping skills		**1**	18% (30)	21% (3)	25% (18)	10% (4)	19% (5)	0% (0)	Supported participants to develop techniques or methods to deal with stressful situations: *“The five-session curriculum focused on the following: (a) exploring personal belief systems*,* especially concerning difficult experiences; (b) understanding the various forms of abuse; (c) understanding and expressing feelings; (d) recognizing healthy relationships; (e) and finding healthy ways to cope with stress.”* (p. 2464, McWhirter, 2011)
9D. Decision making		**2**	7% (12)	14% (2)	10% (7)	3% (1)	7% (2)	0% (0)	Supported participants to identify and assess resolutions to address their specific problems: *“Each unit included skills-based activities that created opportunities to learn and practice conflict management*,* communication*,* and decision-making skills.”* (p. 1484, Feder et al., 2018)
9E. Emotional regulation		**1**	21% (34)	21% (3)	30% (21)	8% (3)	22% (6)	8% (1)	Enhanced the development of healthy strategies to moderate or diffuse negative emotions: *“TARGET teaches a single sequential skill set designed based on research showing that affect regulation involves recognizing*,* modulating*,* and recovering from negative emotion states (Kessler & Staudinger*,* 2009) and accessing and sustaining positive emotion states (Eisner*,* Johnson*,* & Carver*,* 2009)”* (p. 561, Ford et al., 2011)
9 F. Goal setting		**3**	20% (33)	7% (1)	14% (10)	41% (16)	11% (3)	23% (3)	Enabled participants to understand and undergo the process required to achieve a goal: *“Self-efficacy develops as the client is supported in autonomous*,* competent decisions through collaborative goal setting and commitment for change.”* (p. 11, Osterman et al., 2014)
9G. Parenting skills		**2**	54% (89)	36% (5)	66% (47)	28% (11)	63% (17)	70% (9)	Supported parents to develop the skills to provide care, support and protection for their child: *“The overarching framework of TPP strives to enhance adaptive mother-child interaction by creating a relational context that facilitates the toddler’s self-development. By fostering positive affective expression and communication between mother and child and by helping mothers achieve accurate developmental expectations for their child*,* TPP targets the formation of a stronger and more positive relationship.”* (p. 140, Cicchetti et al., 2000)
9 H. Problem solving skills		**2**	28% (46)	29% (4)	38% (27)	13% (5)	19% (5)	39% (5)	Enabled participants to tackle challenging situations and identify effective solutions: *“[listing key elements of the intervention] (3) collaborative problem solving to devise solutions to family challenges”* (p. 299, Fergusson et al., 2013)
9I. Stress management		**2**	22% (36)	29% (4)	30% (21)	13% (5)	19% (5)	8% (1)	Supported the development of methods and techniques to overcome feelings of stress: *“Module 7.* *Managing the stresses of parenting and family life: Identify and coach caregivers on ways to effectively manage household stresses and frustrations”* (Barnhart et al., 2020)
9 J. System navigation		**2**	12% (19)	36% (5)	8% (6)	3% (1)	19% (5)	15% (2)	Supported participants to understand and navigation skills health and social care systems and resources: *“In addition to the safety plan*,* the interviewers provided a list of community resources*,* such as emergency shelter*,* legal aid*,* law enforcement*,* and counseling*,* and strategies for seeking help from these resources. As part of the intervention*,* interviewers also offered to assist women with telephone calls to social service agencies or women’s groups who could act as advocates for abused women.”* (p. 2061, Cripe et al., 2010)
9 K. Financial skills		**2**	4% (6)	14% (2)	0% (0)	3% (1)	7% (2)	8% (1)	Supported the development of understanding of skills related to managing money: *“Improving financial management skills of mothers by reviewing sources of income and expenses*,* and brainstorming methods of increasing income and decreasing expenses utilizing financial management worksheets to reduce stress that often triggers substance use and neglectful behavior”* (p. 8, Donohue et al., 2014)
10. Facilitators to engagement		**1**	27% (44)	29% (4)	31% (22)	33% (13)	11% (3)	15% (2)	Implementation processes which aided or encouraged participant engagement in the intervention: *“A number of adaptations were made for the specific Head Start population. First*,* the groups were conducted in Head Start offices within geographic proximity to the Head Start school sites. We had originally hoped to have groups held at the Head Start centers*,* but many centers were very small with no extra room for groups necessitating having them at the offices. Our primary concern was to overcome the logistical barriers (transportation*,* time*,* other child-care responsibilities) that often hinder clients from participating in therapy.”* (p. 41, Mennen et al., 2021)
11. Increasing knowledge			57% (94)	79% (11)	55% (39)	74% (29)	33% (9)	46% (6)	Educational and informational elements which aimed to increase knowledge or raise awareness.
11 A. Domestic violence and abuse		**2**	16% (26)	71% (10)	13% (9)	8% (3)	7% (2)	15% (2)	Education on elements of DVA and the effects of DVA: *“Topics for session 2 included stress management skills*,* consequences of abuse*,* cycle of abuse*,* and making a safety plan.”* (p. 5, Zlotnick et al., 2011)
11B. Health promotion		**2**	18% (29)	7% (1)	14% (10)	38% (15)	4% (1)	15% (2)	Education on health promotion and health-related behaviours and practices: *“Module 9 (“Life skills”) provides practical advice on diet and nutrition*,* budgeting*,* health care and exercise*,* and so forth when needed.”* (p. 385, Dawe & Harnett, 2007)
11 C. Mental health		**2**	22% (36)	14% (2)	39% (28)	3% (1)	11% (3)	15% (2)	Education on elements of MH and the effects of MH: *“Goals are to educate families about depressive disorders*,* increase family awareness of the impact of stress and depression on functioning”* (p. 7, Compas Bruce et al., 2009)
11D. Substance misuse		**2**	20% (33)	0% (0)	0% (0)	64% (25)	19% (5)	23% (3)	Education on elements of SU and the effects of SU.: *“The manual provided guidance on the risks of substance use*,* the importance of abstinence*,* and the benefit of seeking drug and alcohol treatment outside of the prenatal setting.”* (p. 440, Yonkers et al., 2012)
12. Managing symptoms and relapse prevention		**3**	37% (61)	0% (0)	48% (34)	44% (17)	30% (8)	15% (2)	Management of symptoms of psychological disorders or substance abuse disorders as well as techniques for relapse prevention: *“The BCT sessions were used to (a) help male partners remain abstinent from drugs and alcohol by reviewing and reinforcing compliance with a verbal contract that served to support the male partners’ sobriety on a daily basis”* (p. 419, Kelley & Fals-Stewart, 2002)
13. Post-intervention and long-term support		**3**	26% (42)	43% (6)	17% (12)	21% (8)	26% (7)	69% (9)	Provision of long-term support to participants which included booster sessions to maintain the positive effects of the intervention: *“On average*,* CHWs made six antenatal visits (SD = 3.8)*,* five postnatal visits between birth and 2 months post birth (SD = 1.9)*,* and 1.4 visits/month until the children were 18 months old. After 18 months*,* visits only occurred once every 6 months.”* (p. 717, Rotheram-Borus et al., 2015)
14. Promoting health-related behaviours and development		**1**	37% (60)	21% (3)	31% (22)	51% (20)	26% (7)	62% (8)	Promotion of child health and development and parental health including pre- and post-natal health: *“They followed detailed visit-by-visit guidelines in their efforts to (1) improve the outcomes of pregnancy by promoting women’s healthy prenatal behaviors; (2) improve the health and development of the child by promoting parents’ competent care of their children; (3) enhance parents’ life-course development by encouraging parents to plan subsequent pregnancies*,* complete their education*,* and find work”* (p. 7, Olds et al., 2007)
15. Reflective practice and active feedback		**2**	31% (50)	21% (3)	32% (23)	36% (14)	30% (8)	15% (2)	Reflection and introspection of oneself, behaviours and involvement during the intervention process: *“MIO is a manualized 12-session individual therapy developed to enhance a mother’s capacity for mentalization or reflective functioning (RF) in the parenting role (Suchman & Bers*,* 2015)”* (p. 618, Suchman et al., 2017)
16. Reinforcing positive behaviours		**1**	7% (11)	7% (1)	3% (2)	13% (5)	7% (2)	8% (1)	Positive reinforcement of healthy/ positive behaviours through incentives and praise: *“Contingency management. Abstinent-contingent vouchers ($25 per occasion) were available twice weekly for 22 weeks to participants testing opiate and cocaine negative.”* (p. 170, Jones et al., 2011)
17. Sense of self, self-esteem and self-efficacy		**1**	20% (33)	29% (4)	17% (12)	10% (4)	37% (10)	23% (3)	Supported the development of a healthy concept of self, positive self-efficacy and self-esteem: *“The MEP aims to increase women’s self-efficacy and reduce their self-blame through participation in group discussions and providing group support.”* (p. 260, Clark et al., 2018)
18. Social support		**3**	30% (49)	29% (4)	38% (27)	23% (9)	30% (8)	8% (1)	Provision of social support through the group setting and supporting the development of one’s social support: *“RPMG was developed as a supportive treatment*,* with the use of a group format designed to help women develop their interpersonal skills*,* to perceive the universality of dilemmas pertaining to roles as women and mothers (e.g.*,* Yalom*,* 1985)*,* and to benefit from mutually supportive interpersonal networks.”* (p. 244, Luthar et al., 2007)
19. Stabilisation		**2**	15% (24)	36% (5)	8% (6)	5% (2)	22% (6)	38% (5)	Aimed to provide greater stability to participants lives, giving participants the potential to participate in the intervention: *“Therapists regularly assessed and addressed safety concerns*,* provided emotional support to the mothers*,* assessed families’ current needs (e.g.*,* food*,* transportation*,* etc.)*,* offered referrals and help as indicated*,* and delivered donated goods such as furniture and small household items.”* (p. 709, Jouriles et al., 2009)
20. Tailoring and personalising		**1**	40% (65)	21% (3)	41% (29)	54% (21)	30% (8)	31% (4)	Modification or tailoring of intervention components dependent upon participant characteristics: *“Home visitors discussed each woman’s individual experiences and tailored the intervention to her expressed needs and level of danger at each visit”* (p. 1131, Sharps et al., 2016)

NB. PPIE priority level 1 = first priority, to be provided first; 2 = second priority, to be provided second; 3 = third priority, to be provided third.

### Addressing Additional Needs

Around a fifth of interventions (18%) included a component addressing additional needs, such as those concerning economic, housing or employment needs. These included employment skills training, parental life-skills education (Donohue et al., 2014; Jacobs et al., 2016) and directing families to services supporting basic needs, such as food pantries (Guo et al., 2016). This component did not relate to stabilising an individual prior to the intervention but was related to components which addressed additional needs during the intervention. Addressing additional needs was common in clustered interventions (46%).

### Changing Cognitive Processes

Changing cognitive processes involved intervention elements aimed at changing maladaptive thought processes and perceptions using approaches such as cognitive behavioural therapy or cognitive restructuring. This component was common in dyadic (48%) and MH interventions (37%), with a third of interventions including this component (32%).

### Community Referrals

Around one fifth of interventions (18%) included a community referral component which involved linking participants with local organisations providing support for DVA, MH or SU. Parents were referred to the relevant service dependent upon the detected problem (Bartu et al., 2006). For example, parents or caregivers were referred for mental-health services when deemed appropriate (Boyd et al., 2017). Home visiting interventions commonly included this component, signposting parents to support (Duggan et al., 1999, 2004, 2007; Jack et al., 2019; LeCroy & Krysik, 2011). Community referrals were commonly present in DVA (36%) and clustered interventions (31%).

### Cultural and Community Adaptations

Several interventions (17%) included a component modifying the intervention to reflect different perspectives, needs and practices of specific cultural, ethnic geographic and/or religious communities. For example, intervention materials or content, such as psychoeducation modules, were modified to reflect the intervention population (Jones et al., 2014) or specific practices and customs (Walkup et al., 2009). This component was most prevalent in MH interventions (28%).

### Developing a Safe Intervention Space

#### Intervention Environment

Several interventions (17%) described ways in which providers made considered decisions about the intervention environment to develop a safe and comfortable space for participants. For some this involved in-home delivery of the intervention (Beeber et al., 2013; Black et al., 2003) or group sessions involving those with similar experiences (Rosenblum et al., 2017). This component was most common in DVA interventions (50%).

#### Relationship between Participant and Provider

A third of interventions (34%) described ways in which they had supported the development of a strong and trusting relationship between participant and provider. This included supporting the development of a therapeutic relationship (Graham-Bermann et al., 2019), positive partnerships between family-support workers and participants (Fergusson et al., 2006), or relationships between paraprofessional or peer mentors and participants (Cupples et al., 2011). This component was most commonly present in clustered interventions (54%) followed by dyadic (52%) and DVA interventions (43%).

### Developing Healthy Relationships

#### Family Relationships

Several interventions (23%) included elements aiming to improve relationships within the family within or outside the home. This included identifying the priorities and goals of the family and introducing concepts related to family relationships (Barnhart et al., 2020) or improving family functioning (Duggan et al., 2007; Ondersma et al., 2017). This component was most common in clustered interventions (54%).

#### Parent-Child Relationships

Almost half of interventions (44%) included activities aiming to improve parent-child relationships. These included modelling effect parent-child interaction (Duggan et al., 1999), targeting parenting behaviours related to parent-child bonding (Silovsky et al., 2011) or using restorative parenting to rebuild father-child relationships (Stover, 2015). This component was most common in MH interventions (61%) followed by clustered (54%) and dyadic interventions (48%).

#### Parent Relationships

A few interventions (13%) included activities to improve interparental relationships. For example, interventions provided support for parents to help them address relationship problems (Guo et al., 2016), support to improve interparental communication for co-parenting (Stover, 2015) or broader communication skills (Barlow et al., 2019), as well as psychoeducation to reduce conflict, manage stress and co-parent (Wadsworth et al., 2011). Parent relationships was common in DVA interventions (36%).

### Empowerment

Empowerment described any component focused on supporting an individual to recognise their strengths and capabilities, and take control of their actions and lives (Jones et al., 2014). The empowerment component was most common in DVA interventions (36%) with models such as the empowerment process model (Clark et al., 2018) being used and brief empowerment interventions being added to existing support (Jack et al., 2019).

### Enhancing Motivation To Change

Around a quarter of interventions (26%) involved techniques to encourage and promote motivation to change behaviour, with the aim of reducing or eliminating maladaptive behaviours. Osterman (Osterman, 2009) provides an example of this component within their motivational interviewing intervention: *“The importance and confidence of the woman for changing drinking behaviours was assessed*,* followed by further exploration of the importance of change for the woman and the building of her confidence for change. During this process*,* information regarding strategies for behaviour change was exchanged while resistance for behaviour change was reduced”* (p. 47). This component was most common in SU interventions (51%).

### Enhancing Skills

#### Communication Skills

Around a fifth of interventions (21%) supported participants in developing communication skills, aiming to enable them to talk, share and express their ideas and emotions (e.g., Cicchetti et al., 2000; Feder et al., 2018; Slesnick & Erdem, 2013). This component was most common in DVA interventions (36%).

#### Conflict Resolution

A few interventions (10%) involved a component aiming to help participants understand and undergo the process of finding peaceful solutions to conflict (e.g., Barnhart et al., 2020; Clark et al., 2018; Feder et al., 2018). As above, this component was most common in DVA interventions (50%).

#### Coping Skills

Several interventions (18%) supported participants to learn how to cope with stressful situations. For example, McWhirter et al. (McWhirter, 2011) talk about discussing and working on coping strategies: *“the women were presented with information regarding adaptive and nonadaptive coping strategies. Participants were then encouraged to identify a nonadaptive coping strategy to work on changing as part of the treatment process”* (p. 2464). This component was most common in DVA (21%), MH (25%) and dyadic (19%) interventions.

#### Decision-Making

Only a few interventions included a decision-making skills component (7%) aiming to support participants to gather information and assess potential resolutions, to identify a solution to their problems (e.g., Sharps et al., 2016). This was most common in DVA (14%) and MH (10%) interventions.

#### Emotional Regulation

Around a fifth of interventions (21%) included a component helping participants to develop healthy strategies to regulate negative emotions (e.g., Barlow et al., 2019). This was most common in DVA (21%), MH (30%) and dyadic (22%) interventions.

#### Goal Setting

A fifth of interventions (20%) included activities enabling participants to take steps to set and achieve desired goals (e.g., Catalano et al., 1997; Verduyn et al., 2003). This was most common in SU interventions (41%).

#### Parenting Skills

Over half of interventions (54%) supported parents to develop skills to provide their child with physical care, psychological support and protection. This involved addressing parenting needs (e.g., LeCroy & Krysik, 2011), supporting parent-child interactions through role modelling or promoting parental empathy (e.g., Duggan et al., 2007). Over two-thirds of all clustered interventions (69%) included this component. Parenting skills was also a common component in dyadic (63%) and MH interventions (66%).

#### Problem-Solving Skills

Over a quarter of interventions (28%) included activities aiming to enable participants to handle unexpected situation and challenges, and identify solutions. In some instances, this involved role-modelling problem-solving (e.g., Duggan et al., 1999) or practising problem-solving (e.g., Slesnick & Zhang, 2016). Problem-solving skills was most common in MH (38%) and clustered (38%) interventions.

#### Stress Management

The stress-management component was coded for all interventions that involved supporting the development of methods to overcome feelings of stress. Around a fifth of interventions included this component (22%). Often, interventions focused on supporting parents to manage the stresses of parenting and family life (e.g., McWhirter, 2011). This component most often featured in DVA (29%) and MH (30%) interventions.

#### System Navigation

A few interventions (12%) included a component supporting participants to understand and navigate health and social care services and/or community resources. An example of this can be found in Jack et al. (2019): “*System navigation involves identifying*,* referring*,* and actively facilitating participant access to external domestic violence*,* legal*,* housing*,* or other health or social care services*” (p. 1578). This component was most common in DVA (36%), dyadic (19%) and clustered (15%) interventions.

#### Financial Skills

Six interventions (4%) had a component focused on financial skills, which involved supporting parents to develop skills to manage finances, solve financial problems and understand what financial services and support are available to them. This component was most common in DVA interventions (14%).

### Facilitators to Engagement

Interventions were coded as including a component facilitating engagement if they referred to an implementation process or measure which encouraged participant engagement for example via transportation assistance (Mullins et al., 2004; Olds et al., 2010), childcare (Volpicelli et al., 2000) or flexibility in intervention delivery site (Handley et al., 2017). Just over a quarter of interventions (27%) included this component, being most common in MH (31%) and SU interventions (33%).

### Increasing Knowledge

#### DVA

These interventions aimed to increase knowledge and understanding of different types of DVA, the violence cycle, attributes of healthy/unhealthy relationships and safety in intimate relationships (e.g., Feder et al., 2018; Jack et al., 2019; Sharps et al., 2016). This component was most common in DVA interventions (71%).

#### Health Promotion

Interventions were coded thus if they aimed to increase knowledge and understanding of health in general, including the delivery of health-related information through home visits (e.g., Cupples et al., 2011), manuals or specific modules (e.g., Black et al., 2003). This component was most prevalent in SU interventions (38%).

#### MH

Interventions were coded under this component if they provided education or information on MH, including signs and symptoms of mental illness or skills to promote good mental health. Education on specific mental illnesses such as depression, anxiety, post-natal depression and “baby blues” were common (e.g., Zlotnick et al., 2011). This was most common in MH interventions (39%).

#### SU

Interventions were coded thus if they aimed to increase knowledge and understanding of addiction, the effects of SU on parents and children (including behaviour effects of substances) and of relapse prevention and abstinence (e.g., Lander et al., 2015) This component was most common in SU interventions (64%).

### Managing Symptoms and Relapse Prevention

Over a third of interventions (37%) included a component focused on managing symptoms of MH (i.e. anxiety or depression) or SU disorders, or techniques for relapse prevention. This could involve educational modules on remaining abstinent (e.g., Barlow et al., 2019), managing relapses or practicing refusal skills (e.g., Slesnick & Erdem, 2013). This was most common in SU interventions (44%).

### Post-Intervention and Long-Term Support

Around a quarter of interventions (26%) provided long-term support to participants including booster sessions to maintain positive effects. Some interventions offered family intervention support throughout the pre-school years (Fergusson et al., 2013) while others offered discrete booster sessions (Zlotnick et al., 2019). Post-intervention and long-term support was most common in clustered (69%) and DVA (43%) interventions.

### Promoting Health-Related Behaviours and Development

Over a third of interventions (37%) targeted promoting health-related behaviours for parents and children (child health and development and maternal health including pre- and post-natal health). This included promoting immunisation, vaccinations, safer sex practices and medical check-ups (e.g., Bartu et al., 2006; Donohue et al., 2014; Duggan et al., 2007). This was most common in clustered (62%) and SU (51%) interventions.

### Reflective Practice and Active Feedback

Just under a third of interventions (31%) included a reflective practice and active feedback component encouraging participants to reflect on previous intervention sessions or behaviours to identify hidden patterns and thought processes, as well as understand the participant’s motivation for joining the intervention. For example, one intervention involved aimed to enhance a “*mother’s capacity for mentalization or reflective functioning in the parenting role”* (p. 618, Suchman et al., 2017), while others provided opportunities for participants to reflect on previous sessions to reinforce content (Zlotnick et al., 2011). This component was most often identified in SU (36%) and dyadic (30%) interventions.

### Reinforcing Positive Behaviours

A few interventions (7%) aimed to reinforce healthy/positive behaviours through praise or incentives. These included monetary vouchers for abstinence (Jones et al., 2011) or attending treatment (Morgenstern et al., 2006) or participants receiving verbal recognition, stickers on graphs or certificates for negative urine screening results (Jones et al., 2014). This component was most often present in SU interventions (13%).

### Sense of Self, Self-Esteem, and Self-Efficacy

A fifth of interventions (20%) aimed to develop healthy self-concept, self-efficacy and/or self-esteem as an individual or parent. This component aimed to increase women’s self-efficacy and reduce self-blame (Clark et al., 2018), supporting improvements in mother’s knowledge of child development and child rearing practices (Baker-Henningham et al., 2005). This component was most common in dyadic (37%) and DVA (29%) interventions.

### Social Support

Just under a third of interventions (30%) included social support to help participants through the intervention. This included group therapy and community-based interventions. Clark et al. (2018) provides an example of social support within the Mom’s Empowerment Program (MEP): *“The MEP is community-based in that it was developed in partnership with community agencies and domestic violence shelters… It aims to increase women’s self-efficacy and reduce their self-blame through participation in group discussions and providing social support”* (p. 260, Clark et al., 2018). This component was most common in dyadic (30%) and DVA (29%) interventions.

### Stabilisation

Several interventions (15%) included a component aiming to provide greater stability to participants lives, including ensuring families have appropriate housing and living conditions or safety planning (Graham-Bermann et al., 2019). This component was often associated with ‘crisis management’ and supporting families in times of need (e.g., Duggan et al., 2007; Morgenstern et al., 2006; Slesnick & Erdem, 2013; Suchman et al., 2010). Stabilisation was most prevalent in clustered (38%) and DVA (36%) interventions.

### Tailoring and Personalising

Many interventions (40%) modified and/or tailored intervention components to the needs of individuals, families, groups or communities participating in the intervention. Interventions could be tailored to consider a mother and child *“needs*,* strengths*,* and circumstances”* (p. 920, Sullivan et al., 2002) as well as their individual experiences in relation to DVA, MH, and SU. This component was most common in SU interventions (54%) followed by MH (41%) and clustered (31%) interventions.

#### Effectiveness of Intervention Components

Robust variance meta-regressions did not reveal any effective components, that is no single component was significantly associated with improved DVA/MH/SU outcomes. However, there were several individual components that were associated with adverse DVA/MH/SU outcomes. At post-intervention, addressing additional needs (coefficient=-0.26, 95% CIs=-0.43 to -0.08, *p* = 0.008), post-intervention and long-term support (coefficient-0.22, 95% CIs=-0.43 to -0.004, *p* = 0.046), and stabilisation (coefficient=-0.22, 95% CIs=-0.39 to -0.06, *p* = 0.0112) components were associated with an adverse effect on MH, and empowerment had a negative impact on SU (coefficient=-0.18, 95% CIs=-0.35 to -0.02, *p* = 0.0278). At follow-up, developing healthy relationships components were associated with an adverse effect on MH (coefficient=-0.23, 95% CIs=-0.45 to -0.01, *p* = 0.039) and empowerment was associated with an adverse effect on DVA (coefficient=-0.15, 95% CIs=-0.19 to -0.12, *p* < 0.001) and SU (coefficient=-0.30, 95% CIs=-0.37 to -0.22, *p* < 0.001). Full results are tabulated in Supplementary File 6. Caution is needed interpreting these results given the heterogeneity across study designs and populations.

#### Effectiveness of Intervention Type

We also explored whether intervention type might be associated with intervention effectiveness. Taking each type of intervention in turn: five (36%) DVA interventions were coded as having a beneficial effect on DVA outcomes at post-intervention and one (7%) had a beneficial effect on follow-up outcomes; 18 (25%) MH interventions were associated with beneficial effects on at least one MH outcome at post-intervention and seven (10%) of these went on to have longer-term effects on follow-up outcomes; eight (21%) SU interventions demonstrated effects on post-intervention SU outcomes of which two (5%) had beneficial effects at follow-up, and one (3%) intervention was coded as having mixed impacts on SU outcomes; eight (29%) dyadic interventions were coded as having impacts on at least one outcome and only one (4%) had impacts on two target outcomes at post-intervention (MH and SU); and four (31%) clustered interventions resulted in positive impacts on outcomes, spread across domains (one on DVA, two on MH and one on SU). No clustered interventions had effects across multiple or all outcomes.

Robust variance meta-regression analyses examining the efficacy of interventions targeting two or more issues (i.e., dyadic and clustered interventions) versus single-issue interventions (i.e., DVA, MH or SU interventions) are reported in Table 2. These suggest that interventions targeting two or more issues are *less* likely to be helpful for MH and SU outcomes at post-intervention (co-efficient=-0.23, 95% CIs =-0.42 to -0.03, *p* = 0.02; co-efficient=-0.22, 95% CIs=-0.37 to -0.06, *p* = 0.008) and *less* likely to be helpful for MH at follow-up (co-efficient=-0.22, 95% CIs=-0.43 to -0.003, *p* < 0.05) as compared to single-issue interventions.

**Table 2:** Robust variance meta-regressions for interventions targeting two or more issues versus single-issue interventions

Outcome		Coefficient	95% CIs		*p* value
			Lower	Upper	
Post-intervention					
DVA		– 0.4853	– 1.0403	0.0696	0.0832
	Intercept	0.5767	0.0035	1.1499	0.0489
MH		– 0.2281	– 0.4223	– 0.0338	0.0222
	Intercept	0.2867	0.1360	0.4374	0.0003
SU		– 0.2157	– 0.3714	– 0.0600	0.0077
	Intercept	0.2472	0.1183	0.3761	0.0005
Follow-up					
DVA		– 0.0689	– 0.1401	0.0023	0.0548
	Intercept	0.0500	– 0.0231	0.1230	0.1149
MH		– 0.2186	– 0.4342	– 0.0031	0.0470
	Intercept	0.1663	0.0159	0.3168	0.0312
SU		– 0.0207	– 0.1808	0.1394	0.7888
	Intercept	0.1115	– 0.0208	0.2438	0.0886

#### Patient and Public Involvement and Engagement

Stakeholders contributed to our presentation of the findings and our interpretation of the results, providing us with recommendations for our discussion and future work. The Lived Experience Advisory Group suggested ordering our intervention components in terms of their perceived importance/priority level, using a traffic light system (see Table 1). While they argued all the components were important, they emphasised that certain components must be in place before others (e.g., ensuring the intervention starts with ‘developing a safe intervention space’ before moving onto components like ‘managing symptoms and relapse prevention’). The Lived Experience Advisory Group identified five key service features as particularly critical to effective support: (1) meeting the family where they are at; (2) personalising the support offered to the families; (3) providing a safe space for the family; (4) ensuring long-term support; and (5) adapting provision so that it is culturally appropriate. Service providers agreed that these components were essential, emphasising the need for personalisation, addressing underlying needs, and providing a safe space. While both groups were pleased to see interventions attempting to address families’ multiple needs, they were disappointed with the limited evidence for their effectiveness. The Lived Experience Advisory Group suggested that factors such as the *timing* and *quality* of intervention components may contribute to this lack of impact – factors that are often overlooked in current service provision.

## Discussion

To our knowledge, this is the first ICA of common components across DVA, MH and SU interventions, and the first to explore the components of clustered interventions in this field. These findings are critical for policy and service development. Our systematic review (Allen et al., 2022) found no current ‘best-bet’ interventions to support families experiencing these multiple adversities and signalled a need for interventions to address co-occurring risk. We have built on this review to examine the nature and frequency of different change components included in interventions across domains, to explore the potential for integrated services.

The ICA identified 20 components common to interventions aiming to support families with parental DVA, MH and/or SU. There were no components that were unique to singular focus or clustered interventions, suggesting that different interventions are targeting shared or common risks across domains. Certain components were more frequently employed depending on the need. For example, empowerment was typical in DVA interventions, while interventions focused on changing cognitive processes and managing symptoms and relapse prevention featured regularly in MH and SU interventions, respectively. These components might be considered core or necessary building blocks for services for families with these particular issues, but they are not exclusive to any one concern. In contrast, clustered interventions were more likely to include components addressing additional needs and offering post-intervention/long-term support, which is unsurprising given the complex profiles of families experiencing multiple adversities (Early Intervention Foundation, 2021; HM Government, 2022a).

The effectiveness data are concerning for this sector. While reviews of interventions for these ACEs as singular risks have identified effective responses (e.g., Barlow et al., 2015; McGovern et al., 2018; Nylen et al., 2006; Rizo et al., 2011), there are currently no effective interventions for families experiencing a combination of DVA, MH and SU. While there is no evidence currently to suggest that single-focus interventions are ineffective (or harmful) for families with complex/multiple needs, it is likely that families experiencing DVA in the context of SU, for example, may require a different response to those experiencing DVA alone. In addition, the study did not identify any ‘magic bullets’, that is individual effective components. The data suggest that interventions should respond on multiple levels and that the combination of components may be a critical consideration. Indeed, these data suggest that current approaches to clustered risk have the potential to undermine the effects of some components on MH and SU, although this may in part be due to the severity of cases included in these studies. An alternative conclusion is that provision may be best targeted at a primary intervention focus, while not excluding individuals with comorbid conditions – a common approach in specialist sectors.

Our engagement with those with lived experience has been critical to considering the next steps for service development in the context of these disappointing findings. Stakeholders asserted both the need for interventions addressing multiple needs and the inadequacy of current approaches. This engagement also revealed that it is the quality of delivery and timing of interventions, rather than the content per se, that requires attention. Our experts agreed on the value of the identified intervention components but provided examples of how these have been poorly implemented in current services. They emphasised the importance of five key service features: meeting the family where they are at; personalising the support offered to the families; providing a safe space for the family; ensuring long-term support; and adapting provision so that it is culturally appropriate. Service providers agreed on this importance of these five components. Some of these suggestions (e.g., recognition that support needs to be personalised for each family), chime with UK Government recommendations (HM Government, 2022c).

This work indicates the need for further (re)design and development of services to support families with multiple needs, with a clear ethos of working with families at the intersection of needs, and considering support for parents and children. Moreover, there is a dearth of interventions addressing social and structural determinants, such as poverty, lack of education and discrimination, or wider systemic issues in the aetiology and impact of these adverse experiences. Despite agreement that these factors are central to prevention (e.g., Marmot et al., 2020; The Lancet Public Health, 2021), almost all the programmes we identified focused on individual behaviour change or relational support. More could be done to develop existing components. For example, only one identified intervention (Skar et al., 2021) aimed to influence wider community values or interactions, and two studies (Silverstein et al., 2017; Skar et al., 2021) referred to the potential for system change but these did not feature as formal change components in the interventions. There is scope for integrated care approaches (Baxter et al., 2018; Ross & Greenberg, 2020) to inform the design of new interventions for families with complex needs.

## Limitations and Future Research

This ICA explored the components of interventions identified in our earlier systematic review (Allen et al., 2022). The scope of that review on family-focused interventions prevented conclusions about the nature and effectiveness of intervention components targeting DVA, MH or SU in adults alone (i.e., not as parents), which may be informative for service development in this sector. In the UK and elsewhere, MH and SU services are commonly commissioned and delivered separately for adults and children/young people. Despite calls for more integration (HM Government, 2022b; Stolper et al., 2024), the evidence base largely reflects this siloed provision. Future research could explore whether the components identified in family-focused interventions are mirrored in adult-only programmes and the relative effectiveness of those components.

Our findings are limited by the quality of reporting of intervention descriptions in evaluation papers. Improved reporting standards introduced over the past decade, due to guidelines like the TIDieR checklist (Hoffmann et al., 2014), mean that components included in more recently evaluated interventions are likely to be over-represented in our ICA. Furthermore, poor reporting of intervention programme theories and informing theoretical frameworks limit the extent to which shared mechanisms of change across domains can be inferred. Adopting a realist perspective to future systematic reviews of clustered interventions may provide more useful information about the drivers of change in particular contexts (see Bonell et al., 2023).

Most ICAs, ours included, are limited to assessing the presence/absence of components in interventions and relating this to effectiveness. This type of analysis does not account for variation in how components are delivered, the quality of delivery, or key contextual factors (e.g., practitioner skill, participant engagement or readiness). As a result, the ICA may obscure complex mechanisms of change that rely on synergistic or additive combinations of multiple components. The impact of interventions, and their constituent components, is dependent on myriad implementation and contextual factors (Bauer & Kirchner, 2020). While this study has demonstrated that the presence of any singular component was not related to effects on DVA, MH or SU outcomes, it is plausible that positive impacts for families with complex needs may be achieved via attention to those contextual factors and/or a combination of two or more components. Indeed, the lack of identifiable ‘effective’ components is perhaps unsurprising given that impact is more likely to arise from the interaction between multiple components – a point raised by others (EIF, 2022). Future research could undertake a qualitative comparative analysis to explore this hypothesis (Sutcliffe et al., 2022).

## Conclusions

This study is the first to explore common components of family-focused interventions targeting DVA, MH and/or SU. While it has been possible to identify shared components across target domains, suggesting the potential to offer integrated services or packages of support to families with complex/multiple needs, findings on the impact of these common elements are not encouraging. Policymakers and service providers developing responses to support families will require more rigorous information about the implementation of these interventions and the contextual factors influencing their impact. In addition, there is a critical need for service design innovation to address the social determinants of DVA, MH and SU in local communities, as well as the systemic issues, such as funding/resourcing models, workforce education and awareness, communication and data sharing, which can hinder effective delivery and sustain service siloes.

## Data Availability

All data generated is available in the Supplementary Materials.

## Materials Availability

Our manuscript reports on a secondary data analysis involving an intervention components analysis of pre-existing literature. All pre-existing literature used is referenced in the Supplementary Materials and available online.
